# Regulation of Cellular Diacylglycerol through Lipid Phosphate Phosphatases Is Required for Pathogenesis of the Rice Blast Fungus, *Magnaporthe oryzae*

**DOI:** 10.1371/journal.pone.0100726

**Published:** 2014-06-24

**Authors:** Md. Abu Sadat, Junhyun Jeon, Albely Afifa Mir, Jaeyoung Choi, Jaehyuk Choi, Yong-Hwan Lee

**Affiliations:** 1 Department of Agricultural Biotechnology, Plant Genomics and Breeding Institute, and Research Institute for Agriculture and Life Sciences, Seoul National University, Seoul, Korea; 2 Center for Fungal Pathogenesis, Plant Genomics and Breeding Institute, and Research Institute for Agriculture and Life Sciences, Seoul National University, Seoul, Korea; 3 Center for Fungal Genetic Resources, Plant Genomics and Breeding Institute, and Research Institute for Agriculture and Life Sciences, Seoul National University, Seoul, Korea; University of Wisconsin – Madison, United States of America

## Abstract

Considering implication of diacylglycerol in both metabolism and signaling pathways, maintaining proper levels of diacylglycerol (DAG) is critical to cellular homeostasis and development. Except the PIP_2_-PLC mediated pathway, metabolic pathways leading to generation of DAG converge on dephosphorylation of phosphatidic acid catalyzed by lipid phosphate phosphatases. Here we report the role of such enzymes in a model plant pathogenic fungus, *Magnaporthe oryzae*. We identified five genes encoding putative lipid phosphate phosphatases (*MoLPP1* to *MoLPP5*). Targeted disruption of four genes (except *MoLPP4*) showed that *MoLPP3* and *MoLPP5* are required for normal progression of infection-specific development and proliferation within host plants, whereas *MoLPP1* and *MoLPP2* are indispensable for fungal pathogenicity. Reintroduction of *MoLPP3* and *MoLPP5* into individual deletion mutants restored all the defects. Furthermore, exogenous addition of saturated DAG not only restored defect in appressorium formation but also complemented reduced virulence in both mutants. Taken together, our data indicate differential roles of lipid phosphate phosphatase genes and requirement of proper regulation of cellular DAGs for fungal development and pathogenesis.

## Introduction

Diacylglycerol (DAG) plays crucial roles in cells as a second messenger in lipid-mediated signaling pathway and as an intermediate in lipid metabolism [1], [2]. DAGs is not a single molecular species but a pool of molecules varying with acyl chain length and saturation level [3], [4]. Mammalian cells produce more than 50 different types of DAGs including polyunsaturated, di-unsaturated, monounsaturated or saturated forms [5]. Different DAGs interact with a diverse array of proteins with C1 domain(s) having different specificities and affinities for DAG, leading to remarkable complexity in DAG-dependent cellular processes [6].

Yeast and mammals have two *de novo* pathways for production of DAG [7]. In one pathway, DAG is synthesized from glycerol-3-phosphate and in another pathway, DAG is generated from dihydroxyaceton phosphate. These two precursors produce lysophosphatidic acid (LPA) and phosphatidic acid (PA) through two acylation steps and finally PA is transformed to DAG by the action of lipid phosphate phosphatase (LPP) [8] (Figure S1). In addition to *de novo* pathways, DAGs can be produced in a manner that is highly dependent on extracellular stimulation. Polyunsaturated DAG is generated from phosphatidyl inositol-4-5-bisphosphate (PIP_2_) by the activity of phospholipase C (PLC) through a single step reaction [9]. Alternatively, monounsaturated/saturated DAGs can be generated in a two-step reaction. In the first step, monounsaturated/saturated phosphatidic acid (PA) is produced from phospholipids through the activity of PLD and in the second step, dephosphorylation of PA by the members of LPP family enzyme results in monounsaturated/saturated DAG [10]. Yeast has two different types of the enzymes, lipid phosphate phosphatase (LPP) and diacylglycerol pyrophosphate phosphatase (DPP) [10] to dephosphorylate PA, whereas mammals lack DPP. Both the LPP and DPP are the members of LPP family.

All the pathways except the one involving PLC converge on dephosphorylation reaction of PA, indicating the importance of LPP in lipid metabolism and DAG-mediated signaling pathways. Due to its status as a gateway to DAG production, LPPs have been well studied and documented in diverse organisms ranging from yeast to plant and insects. In *Arabidopsis thaliana*, *AtLPP1* and *AtLPP2* are involved in stress response and regulation of stomatal movement through ABA signaling, respectively [11], [12]. LPP is shown to play important roles in germ cell migration and tracheal development in insect [13], [14]. In yeast, deletion of individual or both lipid phosphate phosphatase (*ScLPP1*) and diacylglycerol pyrophosphate phosphatase (*ScDPP1*) did not show any visible phenotypes compared to wild type strain, but both genes together controlled cellular reservoir of the phosphatidic acid (PA), lysophosphatidic acid (LPA) and diacylglycerol pyrophosphate (DGPP) [10], [15]. Despite the importance of LPP-mediated regulation of cellular DAGs in different organisms, its implication in fungal pathogenesis remains unexplored. Here we set out to investigate the role of LPP encoding enzymes in development and pathogenesis of a model plant pathogenic fungus, *Magnaporthe oryzae*.

*M. oryzae* is a filamentous fungus that causes the rice blast disease. The rice blast disease is one of the most devastating fungal diseases of rice throughout the world [16]. This disease causes 11 – 30% yield losses of the world rice production and is responsible for recurring epidemics throughout South East Asia and South America [17]. Infection by this fungus begins when an asexual spore called conidium germinates following tight adherence to the surface of host plants. Upon recognition of environmental cues such as surface hydrophobicity, the tip of germ tube develops into a dome-shaped, specialized infection structure called an appressoria [18]. Using turgor pressure generated in appressorium, the fungus mechanically rupture the cuticular layer of the plant and gain access to host tissues [19]. Once inside the host cells, the fungus develops ramifying bulbous invasive hyphae that actively grow to result in visible disease lesion, from which massive number of conidia are produced as secondary inoculum [20]–[22].

Due to genetic tractability of rice blast pathogen and rice [23]–[25], signaling transduction pathways involving cAMP, calcium, and MAP kinase have been well documented for infection-related development in this fungus [26]–[31]. Moreover, other studies suggested that DAG plays significant role in pathogenicity leading to appressorium formation in *M*. *oryzae* [32], [33]. Although a number of studies suggested that lipids and their intermediates are implicated in pathogenesis of fungi including *M. oryzae* [34]–[40], genetic analysis on regulation of cellular DAG, a key element of lipid metabolism and signaling has not been carried out.

As the first step to elucidate the biological functions of *LPP* genes in *M*. *oryzae*, we have identified genes encoding LPP family and functionally characterized those genes through gene deletion approach. Analyses of the deletion mutants showed that individual deletion of *MoLPP3* and *MoLPP5* caused defect in appressorium formation and pathogenicity. Exogenous addition of saturated DAG restored both appressorium formation and virulence defect in both mutants, indicating that maintaining DAG homeostasis is required for fungal pathogenesis. Our work shed light on the critical roles of lipid metabolism during fungal pathogenesis.

## Results

### Identification of *LPP* genes in *M. oryzae*

**Figure 1:**
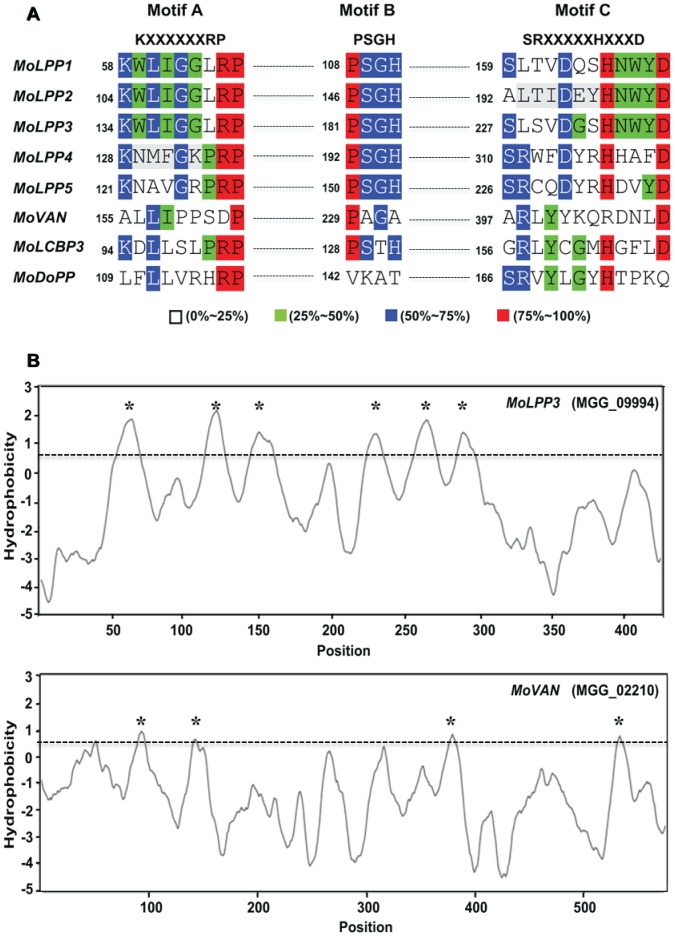
Multiple sequence alignments and Kyte-Doolittle hydropathy plots of PAP2 domain containing proteins in *M. oryzae*. (A) Alignments of three novel sequence motifs among eight PAP2 domain containing proteins using ClustalW algorithm. Different colors indicate the conserved sequence among eight proteins and numbers (left of motifs) show the starting position of three motifs. (B) Hydropathy plots of *MoLPP3* and *MoVAN* proteins showing potential transmembrane domains. Asterisks indicate the potential membrane-spanning domains. Hydropathy plot was generated using TopPred 2 (http://www.sbc.su.se/~erikw/toppred2/). Cutoff value (0.6) was indicated by dotted horizontal line.

To identify the members of PAP2 domain containing LPP encoding genes involved in DAG metabolism in *M. oryzae*, we searched the genome of *M. oryzae* for genes encoding phosphatidic acid phosphatase type 2/haloperoxidase (PAP2) domain (IPR000326), which is a signature of LPP enzymes (http://cfgp.riceblast.snu.ac.kr) [41]. Our search found a total of eight genes encoding PAP2 domain. BLAST search using amino acid sequences encoded by these eight genes as queries showed that five of them were homologous to yeast LPP1 (YDR503C) and DPP1 (YDR284C) (*MoLPP1*; MGG_09330.6, *MoLPP2*; MGG_05988, *MoLPP3*; MGG_09994.6, *MoLPP4*; MGG_05650.6, *MoLPP5*; MGG_12462.6), and the rest of them to vanadium chloroperoxidase (*MoVAN*; MGG_02210.6), long chain base protein 3 (*MoLCBP3*; MGG_17385.67) and dolicylpyrophosphate phosphatase (*MoDoPP*; MGG_09184.6), respectively (Figure S2). Multiple sequence alignment and hydropathy plot revealed that the *MoLPP1* to *MoLPP5*, unlike the rest of PAP2 domain-containing proteins, have conserved three sequence motifs (KXXXXXXRP, PSGH and SRXXXXXHXXXD) (Figure 1A) [42] and six transmembrane domains (Figure 1B and Figure S3), both of which are common features of LPP and DPP enzymes [43]. Based on this data, we only focused on *MoLPP1* to *MoLPP5* in the following analyses. *De novo* synthesis of DAG is known to occur mainly in the endoplasmic reticulum (ER). In accordance with this, PSORT predicted that all *MoLPP*s are localized to ER [44].

### Expression analysis of *MoLPP* genes

**Figure 2:**
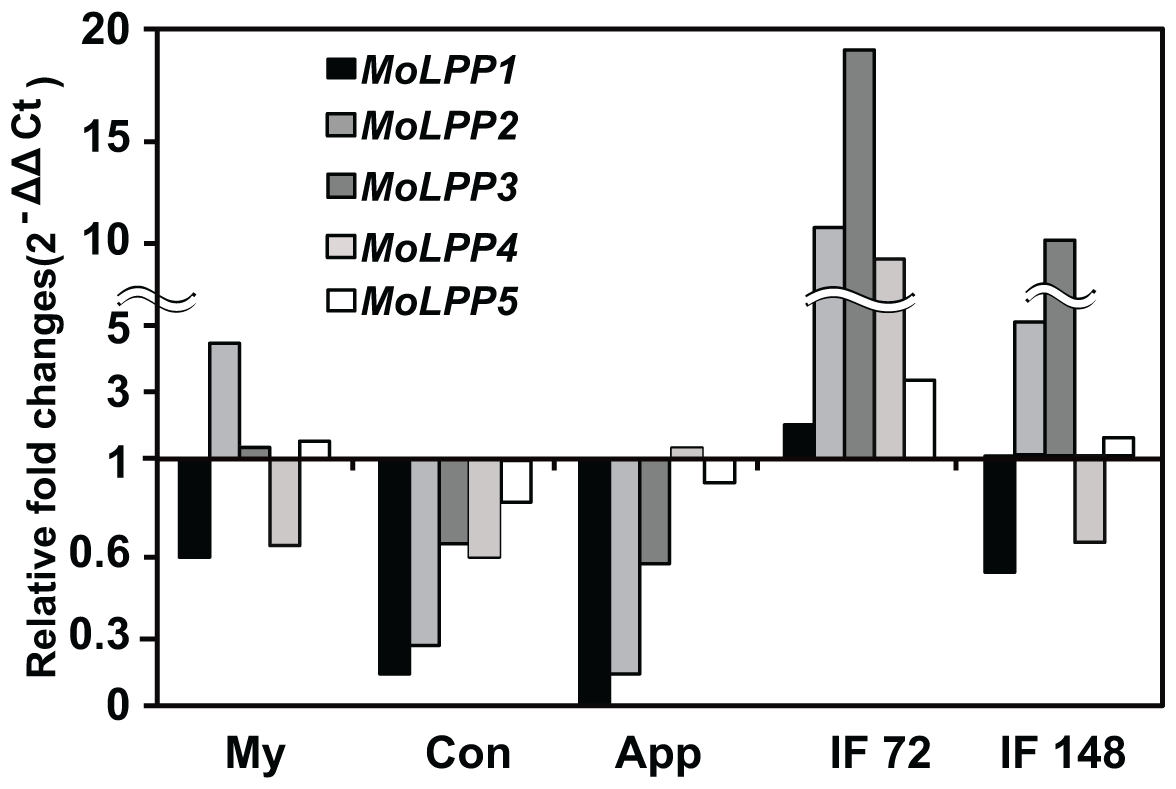
Transcript abundance of *MoLPP* genes in development stages in *Magnaporthe oryzae*. My, mycelia; Con, germinating conidia; App, appressoria; IF 72, early infection stage at 72 hour post inoculation (hpi); IF 148, late infection stage at 148 hour post inoculation (hpi).

As an attempt to infer the implication of *MoLPP* genes in *M*. *oryzae*, qRT-PCR was done with cDNAs sampled from different developmental stages. Expression profiling revealed that the transcript abundance of genes correlates with each other except with *MoLPP4* (Figure 2). Correlation coefficient of *MoLPP4* with expression of other *MoLPP* genes ranged from 0.29 to 0.73, while correlation coefficient for all pairs of other genes was higher than 0.75. This suggests that *MoLPP* genes except *MoLPP4* are likely to share overlapping regulatory mechanisms for transcription. In comparison with germinating conidia, most genes were down-regulated in conidia and appressoria stages. But interestingly, *MoLPP4* was the only up-regulated gene found in appressoria. However, they tended to be up-regulated during host infection. Especially, the expression of *MoLPP2* and *MoLPP3* remained relatively high, compared to the others (Figure 2). In consistent with our observation, the expression of *MoLPP2* and *MoLPP3* genes were detected in *in planta* EST library [45]. We also checked the expression levels of *MoLPP* genes at 40–45 hpi (hours post inoculation) through RNA-Seq data (unpublished data). We found up-regulated expression of *MoLPP3* and *MoLPP5* compare to mycelial stage, whereas the rest three genes were down-regulated at the same stage. Our data suggest the possible involvement of *MoLPP* genes in post-penetration phase of host infection by the rice blast fungus.

### Targeted gene disruption of *MoLPPs*

**Figure 3:**
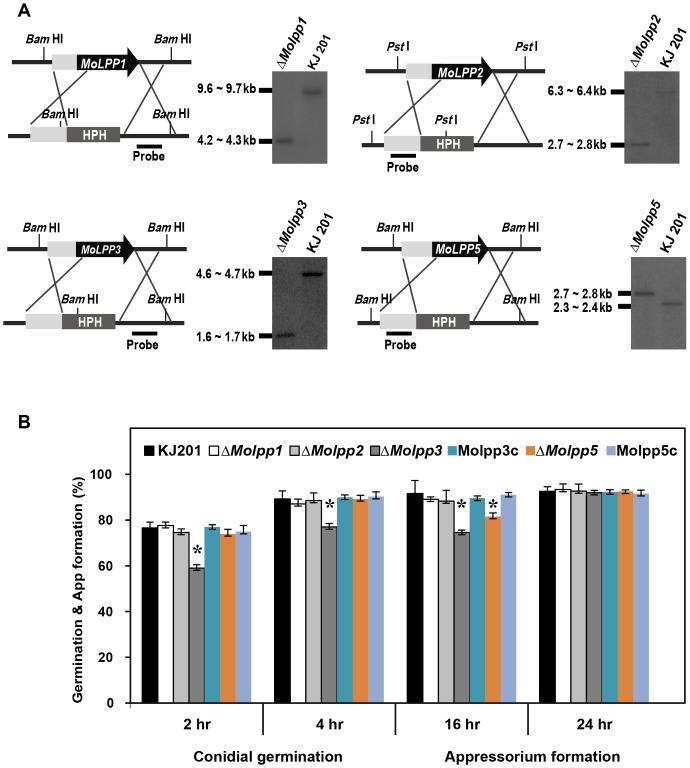
Knock-out mutant generation, conidial germination and appressorium formation. (A) Targeted disruption of *MoLPP1*, *MoLPP2*, *MoLPP3* and *MoLPP5*. Schematic diagram of knockout of target gene (left) and Southern blot analysis of resulting mutant (right) are shown in pair for each gene. (B) Percentage of conidial germination and appressorium formation on hydrophobic surfaces with complemented strains. Asterisks indicate significant difference observed in the mutants, compared to the wild-type (Tukey test, p<0.05).

To investigate the roles of *MoLPP* genes in *M. oryzae*, we generated deletion mutants of individual genes. Knockout constructs were prepared via double joint PCR [46] and directly used for transformation of wild-type protoplasts. Correct gene replacement event in the resulting transformants was confirmed by PCR-based screening and subsequent Southern hybridization analysis (Figure 3A). The deletion mutants were obtained for all but one gene. Despite our repeated efforts, we were not able to generate a deletion mutant for *MoLPP4*. Consequently, we analyzed four genes, of which deletion mutants are available.

### Vegetative growth, germination, and appressorium formation of the mutants

During phenotype analysis, we found that none of the *MoLPP*s were required for vegetative growth and asexual reproduction (Table 1). When the suspensions of mutant spores were incubated on inductive surface, spores of all mutants were capable of germinating and developing appressoria. However, we noticed that both germination and appressorium formation were delayed in *ΔMolpp3* and only - appressorium formation was delayed in *ΔMolpp5* (Figure 3B). Despite - delayed appressorium formation by the two mutants, morphology of appressoria was indistinguishable from that of wild-type. Such delay in germination and appressorium formation in the mutants was complemented by introducing the sequences of *MoLPP3* and *MoLPP5* genes back into the genome of each deletion mutant (Figure 3B).

**Table 1:** Vegetative growth and asexual reproduction of *ΔMolpp1*, *ΔMolpp2*, *ΔMolpp3* and *ΔMolpp5* in *M. oryzae* with complementation.

Strain	Mycelial growth^a^		Conidiation^b^(× 10^4^/ml)
	CM (mm)	MM (mm)	
KJ201	75.8±1.3	70.3±0.6	33.4±5.3
*ΔMolpp1*	76.3±0.6	70.7±0.8	32.2±5.6
*ΔMolpp2*	76.8±0.3	70.3±1.5	33.3±6.3
*ΔMolpp3*	76.3±1.5	70.2±0.8	35.2±5.2
*ΔMolpp3*	76.8±0.8	71.1±1.0	35.3±0.3
*ΔMolpp5*	76.2±0.3	69.5±0.9	31.2±2.9
*Molpp3c*	75.7±0.6	71.5±0.5	32.3±3.5

a Vegetative growth was measured at 12 dpi on complete agar medium and minimal agar medium. Data were presented as mean ± sd from three independent experiments.

b Conidia was measured as the number of conidia from the culture flooded with 5 ml of sterilized distilled water. Data were presented as mean ± sd from three independent experiments.

### Requirement of *MoLPP3* and *MoLPP5* for full virulence of the fungus

**Figure 4:**
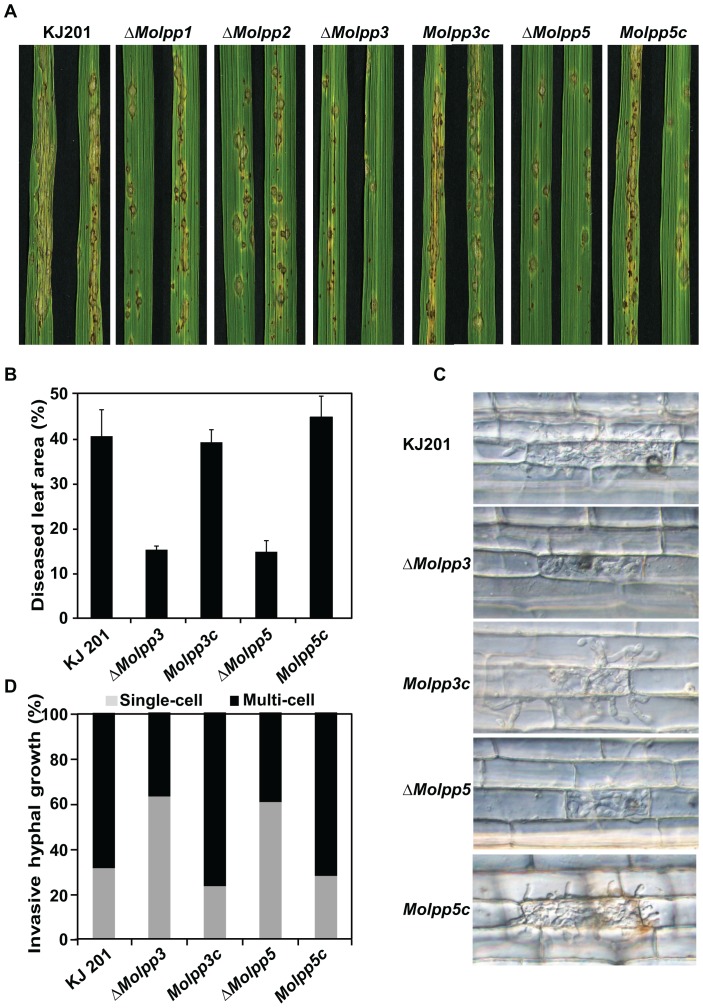
Pathogenicity assay of knockout mutants. (A) Disease development after spraying conidial suspension onto rice leaves. Conidia (1×10^5^/ml) was sprayed onto the leaves and incubated for 7 days. (B) Comparison of diseased leaf area (DLA). The DLA was calculated relative to the total leaf area using the Axiovision image analyzer. (C) Invasive hyphal growth through sheath assay. Rice sheath was injected with 2×10^4^ conidia/ml and observed under microscope at 48 hour post inoculation (hpi). (D) Quantification of invasive growth. Number of cells that are invaded by the fungus was counted using sheath inoculation at 48 hour post inoculation (hpi).

Next, we asked if *MoLPP* genes are involved in fungal pathogenesis on host plants as suggested by the expression analysis. To test this, conidial suspensions of the mutants were spray-inoculated onto rice plants of a susceptible cultivar, Nakdongbyeo. Pathogenicity test showed that *ΔMolpp1* and *ΔMolpp2* were comparable to the wild type in their ability to cause disease on rice plants, whereas *ΔMolpp3* and *ΔMolpp5* were not able to produce as large and many number of disease lesions on rice leaves as the wild-type (Figure 4A). Measurable and significant reduction in number and size of disease lesions were observed for the two mutants, resulting in 62 to 65% reduction in diseased leaf area (DLA), compared to the DLA calculated for leaves inoculated with wild-type strain (Figure 4B). The complementation strains of *ΔMolpp3* and *ΔMolpp5* were as virulent as the wild-type, indicating that *MoLPP3* and *MoLPP5* are required for full virulence of the fungus.

Defect in virulence observed for *ΔMolpp3* and *ΔMolpp5* could not be accounted for by minor delay in appressorium formation. Therefore, we monitored early infection process using sheath inoculation method [47]. The mutant appressoria were able to penetrate plant cells as efficiently as the wild-type appressoria. However, invasive hyphae of both *ΔMolpp3* and *ΔMolpp5* were largely restricted to the primary infected cells at 48 hour post inoculation (hpi) in contrast to invasive hyphae of wild type actively growing to fill in the first host cell and moving to neighboring cells. (Figure 4C and 4D). This result indicates that *MoLPP3* and *MoLPP5* are necessary for the fungus to grow inside host plants in early infection stage.

To date, one of the major causes of defective invasive growth is sensitivity to or inability of the fungus to scavenge/suppresses reactive oxygen species (ROS) that are produced by host plants as a defense response during host-pathogen interaction [48], [49]. In order to test this possibility, we examined sensitivity of the fungus to oxidative stress in the form of H_2_O_2_ or methyl viologen (MV). On complete media containing varying concentration of H_2_O_2_ and MV, radial growth of both mutants was comparable to that of wild-type, suggesting that defect in invasive growth is not ROS-dependent (Figure S4A). In addition, we also checked the sensitivity of the fungus to a cell wall perturbing agent, congo red (CR) but no significant differences were detected between the wild-type and the mutants (Figure S4B).

### Complementation of *ΔMolpp3* and *ΔMolpp5* by DAG

**Figure 5:**
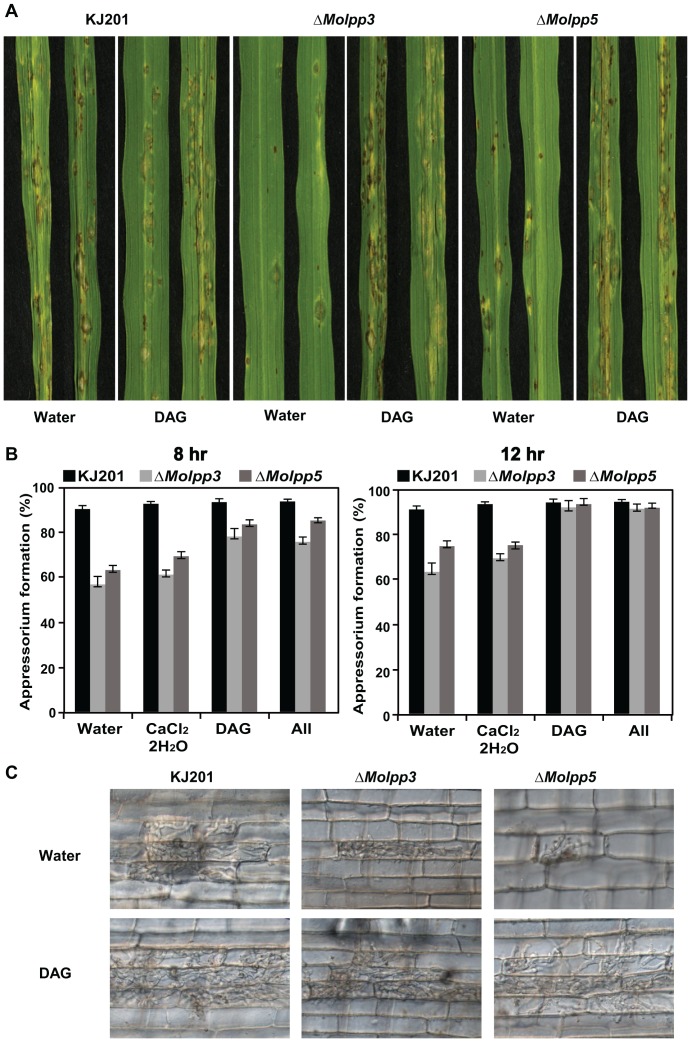
Restoration of appressorium formation and pathogenicity by addition of DAG. (A) Appressorium formation of wild-type and mutants in the presence of DAG and/or CaCl_2_. Conidial suspension (2×10^4^ conidia/ml) was placed the hydrophobic side of cover slips and mixed with DAG and/or CaCl_2_ to final concentrations of 20 µg/ml and 10 mM, respectively. Appressorium formation was observed under a microscope 8 and 12 h after incubation. (B) Spray inoculation (upper panels) and sheath assay (lower panels) with conidial suspensions supplemented with 20 µg/ml of DAG.

Since LPPs are involved in production of DAG using lipids as substrates, we reasoned that imbalance in cellular reservoir of DAG underlies in both pre-penetration and post-penetration defects observed in the mutants. It is known that LPPs are implicated in production of monounsaturated/saturated DAG, whereas PI-PLC-mediated pathway is responsible for polyunsaturated DAG [9]. To test such imbalance in the mutants, we exogenously added saturated DAG (1,2-dicotnly-sn-glycerol: DOG, Sigma-Aldrich) in the conidial suspension and checked appressorium formation at different time points. We also checked appressorium formation after adding CaCl_2_.2H_2_O alone or combination of DAG and CaCl_2_.2H_2_O to examine whether calcium signaling pathway is implicated. We found that addition of saturated DAG was able to restore appressorium formation without delay in the mutants, while CaCl_2_.2H_2_O had little effect in isolation or combination with DAG (Figure 5A). More importantly, when conidial suspensions of the mutants supplemented with DAG were used for spray-inoculation onto rice plants, virulence of both *ΔMolpp3* and *ΔMolpp5* were recovered to the wild-type level (Figure 5B). These data suggest that *MoLPP3* and *MoLPP5* are involved in regulation of cellular DAG and such regulation is important for fungal pathogenesis.

### Transcriptional expression pattern of genes involved in cellular DAG production

**Figure 6:**
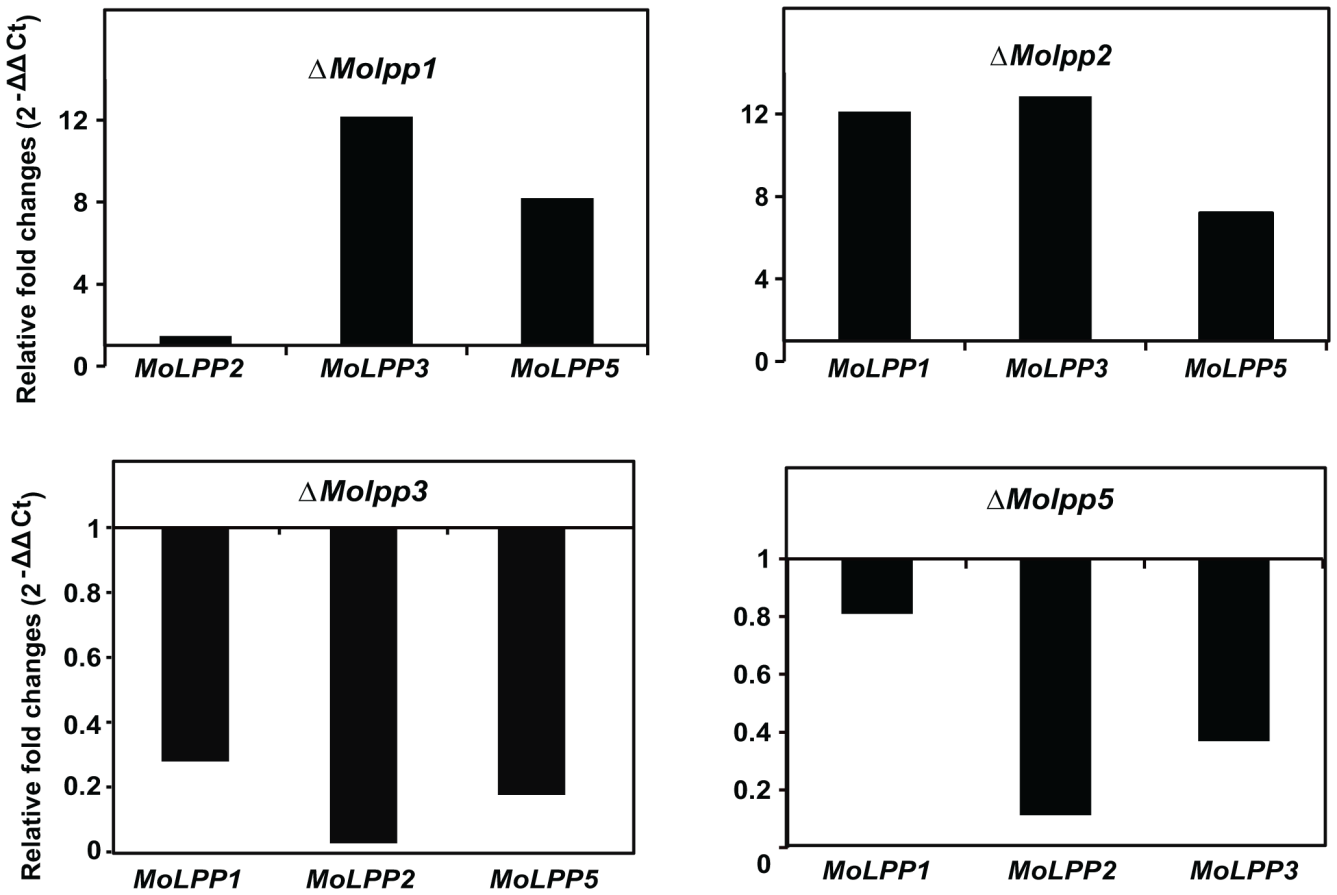
Transcriptional profiling of *MoLPPs* in knockout mutant background. In each knockout mutant, relative transcript abundance of genes was measured for *MoLPP* genes

Given the different phenotypic consequences of deletion of *MoLPP* genes, we investigated the relationship among the genes by checking the transcript abundance of genes in each deletion mutant background. Our transcript analysis showed that deletion of *MoLPP1* or *MoLPP2* increased the expression of rest of the genes, whereas deletion of *MoLPP3* or *MoLPP5* decreased the expression of other genes (Figure 6). It appeared that depletion of *MoLPP1* or *MoLPP2* can be complemented by elevating the expression of *MoLPP3* and/or *MoLPP5* but the reverse could not be achieved. These data suggest that transcriptional regulations of LPP genes are intertwined in a network where expressions of other genes are dependent on expression of *MoLPP3* or *MoLPP5*.

Although PI-PLC-mediated pathway is known to produce different type of DAGs in mammals, we could not exclude the possibility that this pathway contributes to maintaining homeostasis by responding to perturbation in cellular reservoir of DAG. However, when we checked the expression of three PLC encoded genes (*MoPLC1*, *MoPLC2* and *MoPLC3*) in *ΔMolpp3* and *ΔMolpp5* background, none of them showed significant difference relative to the wild type strain, suggesting that *MoLPP*-dependent and PI-PLC-mediated pathways are under independent regulation (Figure S5). Therefore, we rule out the possibility that depletion of cellular DAG level resulted from deletion of LPP genes can be complemented by up-regulation of the alternative pathway.

## Discussion

In many pathogenic fungi, it has been shown that DAG acts as a second messenger involved in regulation of developmental processes as a part of PLC-mediated signaling pathways [29], [50]–[52]. However, roles of DAG are not limited to a second messenger but as diverse as a basic component of membranes, a precursor in lipid metabolism and a central element in lipid-mediated signaling pathway [53]. Such a broad implication of DAG in cellular processes implies that cellular DAG level should be tightly regulated. A critical point at which DAG production and clearance are regulated is the dephosphorylation of PA by LPP.

Here we identified LPP encoding genes using three criteria: presence of PAP2 domain, conservation of three sequence motifs, and presence of six trans-membrane domains in gene-coded proteins. Among the eight genes encoding PAP2 domain, the five that meet the rest of criteria, were selected as candidate LPP genes in *M. oryzae* and named *MoLPP1* to *MoLPP5*. The fact that three distinct motifs and six trans-membrane domains are the hallmark properties of LPP enzymes, suggesting that *MoLPP1* to *MoLPP5* are likely to be bona fide LPPs. It is not clear whether or not *MoVAN*, *MoLCBP3*, and *MoDoPP* have phosphatase activity, considering poor conservation of three motifs that are important for catalytic activity. Among the five putative LPPs, *MoLPP4* and *MoLPP5* were more closely related to DPP1 than LPP1 in *S. cerevisiae*, suggesting that their substrates may include DGPP (Figure S2). Of note, *MoLPP4* seemed to be particularly highly divergent from the yeast LPP1. In conjunction with that, the expression profile of *MoLPP4* deviating the most from the other genes suggests sub-functionalization of *MoLPP4*. It is currently difficult to further address the implication of such divergence due to our failure to generate a knockout mutant strain for the gene.

One possibility of the failure in generation of mutant lacking *MoLPP4* is lethality of gene deletion. However, based on the studies of yeast LPP1 and DPP1, together with our own work, it is not likely that deletion of individual LPP genes including *MoLPP4* is lethal. In *M. oryzae*, as with the other filamentous fungi, it is known that efficiencies of gene deletion by homologous recombination are generally low and dependent on the locus [54], [55]. Therefore, we presume that the genomic environment of the locus harboring *MoLPP4* is not favorable for homologous recombination to occur.

Among the remaining four LPP genes, deletion of either *MoLPP3* or *MoLPP5* resulted in the fungus that was significantly impaired in pathogenesis, in contrast to deletion of *MoLPP1* or *MoLPP2* having no effects on fungal developments and pathogenicity. This discrepancy in effect of deletion between two sets of genes could be explained by our expression analysis that revealed complex regulatory relationship among LPP genes. Since LPPs are not able to directly regulate expression of genes, the nature of such regulatory relationship should be dependent on lipid signaling. Removal of terminal phosphomonoester group from bioactive lipid molecules such as phosphatidic acid through LPP activities is known to result in functional inactivation of these lipids, which otherwise have multitude of effects on cells through interaction with their targets [56]. We conjecture that each LPP might have different but overlapping substrate specificity and that regulation of bioactive lipids by different LPPs would underlie the observed transcriptional network among LPP genes.

Notably, effect of deletion of *MoLPP3* or *MoLPP5* was specific to plant infection. This may be possibly due to functional divergence among *MoLPP* genes after duplication events leading to paralogs in the genome. However, it is still possible that *MoLPP*s function in combinations, making it difficult for us to evaluate their functions independently of other genes via deletion of individual genes. For example, deletion of *MoLPP3* together with other *MoLPP*(s) may reveal roles of *MoLPP3* in vegetative growth. Unfortunately, however, introducing more than three targeted mutations into the fungal genome is technically challenging and our efforts to make double deletion mutant for *MoLPP3* and *MoLPP5* genes were unsuccessful.

We showed that virulence defect of *ΔMolpp3* and *ΔMolpp5* are attributed to the reduced ability of the mutant to grow inside host cells. However, both mutants were comparable to the wild-type in sensitivity to reactive oxygen species and a cell wall perturbing agent, leaving an open question of why invasive growth is significantly impaired in the mutants. In a human pathogen, *Cryptococcus neoformans*, DAG produced by inositol phosphorylceramide (IPC) synthase is known to regulate production of melanin and antiphagocytic protein, two important virulence factors, in at least two ways [52]. First, DAG can bind to C1 domain of Pkc1 leading to increase in Pkc1 activity, which in turn mediates melanin production. The second way is binding of DAG to the transcription factor Atf2 that promote production of antiphagocytic protein 1. Although the genome of *M. oryzae* does not encode an orthologs of IPC synthase, it may be possible that LPP-mediated production of DAG may have an impact on regulation of some virulence factors in *M. oryzae* through its interaction with kinases or transcription factors as shown in *C. neoformans*.

Alternative possibility is related to the effects of LPP activities on the membrane lipid bilayer through production of DAG. Under equilibrium conditions, DAG contributes no significant proportion of cell membranes. However, local and transient accumulation of DAG in the membranes may lead to changes in physical properties of membrane itself and membrane-associated proteins, influencing important cellular processes such as membrane trafficking and exocytosis [57], [58]. In addition, production of DAG will alter lipid compositions in the membranes as well, since DAG is a byproduct of dephosphorylation reaction of lipids. In many intracellular pathogens of animals, lipid rafts, sterol and sphingolipid-rich membrane microdomains, have been shown to control their virulence [59]. Even in human pathogenic fungi, potential role of such membrane domain in pathogenesis was alluded. For example, *Candida albicans* was shown to produce many GPI-anchored virulence factors that are induced along with sterol-rich domain (SRD) [60]. Similarly, it was suggested that membrane localization and release of the virulence factors such as superoxide dismutase and phospholipase B are regulated by special membrane domains in *C. neoformans* [61]. Our expression data shows specific up-regulation of *MoLPP3* and *MoLPP5* during interaction with the host plants. Considering the impact of DAG production on the membranes, it is tempting to speculate that *in planta* specific activities of LPPs may enable the fungus to modulate or remodel physical properties and lipid composition of membranes for successful colonization of rice plants.

In this study, we investigated how regulation of cellular DAG levels is implicated in development and pathogenesis of the plant pathogenic fungus through deletion of genes that encode proteins involved in a key step of DAG biosynthesis. Our results demonstrated that proper regulation of DAG is pivotal for fungal pathogenesis, independently of the PIP_2_-PLC pathway. In particular, we showed that LPP-mediated DAG production has profound impact on invasive growth, during which the most intimate interaction with the host plant occurs. Currently other cellular targets of DAG than PKC1 are not known in *M. oryzae*. Furthermore, there are growing bodies of evidences that suggest the importance of membrane functions in fungal pathogenesis. In light of this, we believe that our work will not only illuminate the importance of DAG production in virulence of plant pathogenic fungi but also have many ramifications on studies regarding lipid metabolisms and membrane-associated processes in fungal pathogenesis.

## Materials and Methods

### Fungal isolates and culture conditions

*M. oryzae* wild type strain KJ201 was obtained from the Centre for Fungal Genetic Resources (CFGR: http://cfgr.snu.ac.kr) for this study. All strains used in this study were grown on V8 agar [V8; 8% V8 juice (v/v), 1.5% agar (w/v), adjusted to pH 6.0 using NaOH] or oatmeal agar [OMA; 5% oatmeal (w/v), 2% agar (w/v)] at 25°C in constant light to promote conidial production [62]. All strains cultured on V8 juice agar media for 7 days or on OMA for 10 to 15 days at 25°C under the continuous light condition to observe the developmental and morphologic phenotypes and pathogenicity ability.

### Nucleic acids manipulation and expression analysis

Fungal genomic DNA was isolated for two purposes using two different methods. For Southern DNA hybridization, genomic DNA was isolated from mycelia according to a standard protocol [63]. For PCR-based screening of transformants, genomic DNA was extracted by quick and easy method [64]. Southern DNA hybridization was performed on selected transformants to ensure correct gene replacement events and absence of ectopic integration. Genomic DNAs were digested with *Pst*l, *Bam*HI and blots were probed with 1-kb 5′-flanking/3′ flanking sequences (Figure 3A). Southern DNA hybridization was done through a standard method [65]. To perform the expression analysis by quantitative real-time PCR (qRT-PCR), cDNA synthesis was performed with 5µg of total RNA using the oligo dT primer with the ImProm-IITM Reverse Transcription System kit (Promega, Madison, WI, USA) following the manufacturer's instruction. Primer pairs used are listed in Table S1. To compare the relative abundance of *MoLPPs* (1–5) transcripts, average threshold cycle (Ct) was normalized to that of *β-tubulin* gene and germinating conidia for each sample.

### Targeted deletion of genes and complementation experiments

Targeted deletions of genes were carried out by transforming wild-type protoplast with knockout constructs. Knockout construct of individual genes were obtained by double-joint PCR where ∼1 kb flanking sequences of target gene were amplified and fused to hygromycin cassette. The resulting transformants were subjected to PCR-based screening and gene replacement event in the genome of transformants were confirmed by Southern blot analysis using one of flanking sequences as a probe. Complemented strains for two mutants (*ΔMolpp3 and ΔMolpp5*) were generated by reintroducing 1.2 kb and 0.9 kb fragments carrying the *MoLPP3* and *MoLPP5* ORF, respectively. Complemented strains were selected through PCR screening with specific primers (MoLPP3_NF, MoLPP3_NR, MoLPP5_NF and MoLPP5_NR) (Table S1) and also used “c” to indicate complementation strain through the whole manuscript.

### Developmental phenotype assay

Radial mycelial growth rate with three replications was measured on complete agar media and on minimal agar media 12 days after inoculation. For conidial germination and appressorium formation, conidia were harvested from 7-days-old V8 juice agar plates and passed through the two layer of miracloth with sterilized distilled water. Concentration of conidial suspension was adjusted to 2×10^4^ conidia per mililiter and 40µl was dropped onto coverslips with three replicates and incubated in moistened box at room temperature. At two and four hours after incubation, germination rate was determined by observing at least a hundred of conidia per replicate under a microscope. The rate of appressorium formation was measured as the percentage of germinated conidia that developed appressoria at 8, 16 and 24 hours each after incubation. These assays were performed with three replicates in three independent experiments. For conidiation, conidia was collected from 7-days-old V8 juice agar plates with 5 ml sterilized distilled water and measured by counting the number of asexual spores within 10µl conidia suspension onto haemacytometer under a microscope.

### Pathogenicity assay and infectious growth visualization

For spray inoculation, conidia were collected from 7-days-old V8 juice agar media with 10 ml of filtered conidia suspension and adjusted to 1×10^5^ conidia per mililiter containing Tween 20 (250 ppm final concentration) and sprayed onto the rice seedlings (*Oryza sativa* cv. Nakdongbyeo) in three to four leaf stage. Inoculated rice seedlings were placed in a dew chamber for 24 hours under the dark condition at 25°C. After then, they were transferred to the rice growth incubator that is maintained at 25°C, 80% humidity and with photoperiod of 16 hours using fluorescent lights [66]. For microscopic observation of invasive growth on rice tissue, excised rice sheath of Nakdongbyeo were prepared by standard process [47]. Conidia suspension was injected in excised rice sheaths and incubated in moistened box for 24 and 48 hours at room temperature. After incubation, the infected rice sheaths were trimmed to remove chlorophyll enriched plant parts. Remaining epidermal layers of mid vein (three to four cell layers thick) were used for microscopic experiment.

### Computational analysis

All sequence information used in this study was taken and analyzed from the online database Comparative Fungal Genomics Platform (CFGP) [41], http://cfgp.snu.ac.kr/) and BLAST program provided at the National Center for Biotechnology Information, Bethesda, USA (http://www.ncbi.nlm. nih.gov/blast/) [67]. Sequences were aligned by ClustalW algorithm [68]. Hydropathy plot was generated using TopPred 2 (http://www.sbc.su.se/~erikw/toppred2/) [69].

### Tests for sensitivity to reactive oxygen species and a cell wall perturbing agent

Sensitivity to reactive oxygen species was tested by growing fungi on complete media supplemented with H_2_O_2_ and methylviologen. Test for cell wall integrity was carried out by adding a cell wall perturbing agent, Congo red (200 ppm) to complete media that are inoculated with wild-type and mutant strains.

## Supporting Information

Figure S1 **The enzymatic reactions leading to DAG generation through different pathway.** D P, dihydroxyaceton phosphate; D P Acyl, dihydroxyaceton phosphate acyltransferase; 1-Acyl-D P, 1-Acyl-dihydroxyaceton phosphate; 1-Acyl-D P Red, 1-Acyl-dihydroxyaceton phosphate reductase; G-3-p, glycerol-3-phosphate; G-3-p Acyl, glycerol-3-phosphate acyltransferase; Lyso PA; lysophosphatidic acid; PC, phosphatidylcholine; PLD, phospholipase D; PA, phosphatidic acid; PIP_2_, phosphatidyl inositol-4-5-bisphosphate; PLC, phospholipase C; DAG, diacylglycerol.
(TIF)

Figure S2 **Sequence similarities of PAP2 domain of eight genes with each other and also with the yeast** ***ScLPP1*** **and** ***ScDPP1*** **.** Sequence similarities were measured by using BLAST2 with the amino acid sequences provided at Comparative Fungal Genomics Platform (CFGP), (http://cfgp.snu.ac.kr/).
(TIF)

Figure S3 **Hydropathy plots of the deduced** ***MoLPP1*** **,** ***MoLPP2*** **,** ***MoLPP4*** **,** ***MoLPP5*** **,** ***MoLCBP3*** **and** ***MoDoPP*** **proteins showing potential transmembrane domains (** ***TM1-6*** **).** Asterisks are indicating the potential number of membrane-spanning domains. Hydropathy plot was generated using TopPred 2 (http://www.sbc.su.se/~erikw/toppred2/). Cutoff value (0.6) was indicated by dotted horizontal line.
(TIF)

Figure S4 **Mycelial growth in different stress conditions.** (A) Oxidative stress (B) Congo red. Mycelial block was inoculated in CM agar plate containing different concentration of hydrogen peroxide, methyl viologen and congo red. Data was taken at 10 dpi with three independent experiments with three replications.
(TIF)

Figure S5 **Transcriptional profiling of phospholipase genes of** ***M. oryzae*** **in** ***ΔMolpp3*** **and** ***ΔMolpp5*** **knockout mutants.** Transcriptional expressions of *MoPLC* genes in knock-out mutants were compared with the expression of wild type strain following normalization using β-tubulin.
(TIF)

Table S1 **Oligo sequences used in this study.**
(DOCX)

## References

[1] Bell RM, Coleman RA (1980) Enzymes of glycerolipid synthesis in eukaryotes. Annu Rev Biochem 49: 459–487. 6250446 10.1146/annurev.bi.49.070180.002331

[2] Nishizuka Y (1984) The role of protein kinase C in cell-surface signal transduction and tumor promotion. Nature 308: 693–698. 6232463 10.1038/308693a0

[3] Deacon EM, Pettitt TR, Webb P, Cross T, Chahal H, et al (2002) Generation of diacylglycerol molecular species through the cell cycle: a role for 1-stearoyl, 2-arachidonyl glycerol in the activation of nuclear protein kinase C-beta II at G2/M. J Cell Sci 115: 983–989. 11870217 10.1242/jcs.115.5.983

[4] Pettitt TR, Martin A, Horton T, Liossis C, Lord JM, et al (1997) Diacylglycerol and phosphatidate generated by phospholipases C and D, respectively, have distinct fatty acid compositions and functions. Phospholipase D-derived diacylglycerol does not activate protein kinase C in porcine aortic endothelial cells. J Biol Chem 272: 17354–17359. 9211874 10.1074/jbc.272.28.17354

[5] Pessin MS, Raben DM (1989) Molecular-species analysis of 1,2-diglycerides stimulated by α-thrombin in cultured fibroblasts. J Biol Chem 264: 8729–8738. 2542285

[6] Wakelam MJO (1998) Diacylglycerol - when is it an intracellular messenger? Biochim Biophys Acta 1436: 117–126. 9838074 10.1016/s0005-2760(98)00123-4

[7] Athenstaedt K, Daum G (1999) Phosphatidic acid, a key intermediate in lipid metabolism. Eur J Biochem 266: 1–16. 10542045 10.1046/j.1432-1327.1999.00822.x

[8] Nanjundan M, Possmayer F (2003) Pulmonary phosphatidic acid phosphatase and lipid phosphate phosphohydrolase. Am J Physiol Lung Cell Mol Physiol 284: L1–L23. 12471011 10.1152/ajplung.00029.2002

[9] Nishizuka Y (1992) Intracellular signaling by hydrolysis of phospholipids and activation of protein kinase C. Science. 258: 607–614. 10.1126/science.1411571 1411571

[10] Toke DA, Bennett WL, Dillon DA, Wu WI, Chen XM, et al (1998) Isolation and characterization of the *Saccharomyces cerevisiae* DPP1 gene encoding diacylglycerol pyrophosphate phosphatase. J Biol Chem 273: 3278–3284. 9452443 10.1074/jbc.273.6.3278

[11] Paradis S, Villasuso AL, Aguayo SS, Maldiney R, Habricot Y, et al (2011) *Arabidopsis thaliana* lipid phosphate phosphatase 2 is involved in abscisic acid signalling in leaves. Plant Physiol Biochem 49: 357–362. 21277215 10.1016/j.plaphy.2011.01.010

[12] Pierrugues O, Brutesco C, Oshiro J, Gouy M, Deveaux Y, et al (2001) Lipid phosphate phosphatases in *Arabidopsis*. Regulation of the AtLPP1 gene in response to stress. J Biol Chem 276: 20300–20308. 11278556 10.1074/jbc.M009726200

[13] Ile KE, Tripathy R, Goldfinger V, Renault AD (2012) Wunen, a *Drosophila* lipid phosphate phosphatase, is required for septate junction-mediated barrier function. Development 139: 2535–2546. 22675212 10.1242/dev.077289

[14] Zhang N, Zhang JP, Cheng Y, Howard K (1996) Identification and genetic analysis of wunen, a gene guiding *Drosophila melanogaster* germ cell migration. Genetics 143: 1231–1241. 8807296 10.1093/genetics/143.3.1231 PMC1207393

[15] Toke DA, Bennet WL, Oshiro J, Wu WI, Voelke DR, et al (1998) Isolation and characterization of the *Saccharomyces cerevisiae* LPP1 gene encoding a Mg^2+^-independent phosphatidate phosphatase. J Biol Chem 273: 14331–14338. 9603941 10.1074/jbc.273.23.14331

[16] Talbot NJ (2003) On the trail of a cereal killer: Exploring the biology of *Magnaporthe grisea* . Annu Rev Microbiol 57: 177–202. 14527276 10.1146/annurev.micro.57.030502.090957

[17] Baker B, Zambryski P, Staskawicz B, Dinesh-Kumar SP (1997) Signaling in plant-microbe interactions. Science 276: 726–733. 9115193 10.1126/science.276.5313.726

[18] Howard RJ, Ferrari MA, Roach DH, Money NP (1991) Penetration of hard substrates by a fungus employing enormous turgor pressures. Proc Natl Acad Sci U S A 88: 11281–11284. 1837147 10.1073/pnas.88.24.11281 PMC53118

[19] Bourett TM, Howard RJ (1990) Invitro development of penetration structures in the rice blast fungus *Magnaporthe grisea* . Can J Bot 68: 329–342.

[20] Balhadere PV, Foster AJ, Talbot NJ (1999) Identification of pathogenicity mutants of the rice blast fungus *Magnaporthe grisea* by insertional mutagenesis. Mol Plant Microbe Interact 12: 129–142.

[21] Kankanala P, Czymmek K, Valent V (2007) Roles for rice membrane dynamics and plasmodesmata during biotrophic invasion by the blast fungus. Plant Cell 19: 706–724. 17322409 10.1105/tpc.106.046300 PMC1867340

[22] Urban M, Bhargava T, Hamer JE (1999) An ATP-driven efflux pump is a novel pathogenicity factor in rice blast disease. EMBO J 18: 512–521. 9927411 10.1093/emboj/18.3.512 PMC1171144

[23] Dean RA, Talbot NJ, Ebbole DJ, Farman ML, Mitchell TK, et al (2005) The genome sequence of the rice blast fungus *Magnaporthe grisea* . Nature 434: 980–986. 15846337 10.1038/nature03449

[24] Goff SA (2005) A draft sequence of the rice genome (*Oryza sativa* L. ssp. *japonica*). Science 309: 879–879. 11935018 10.1126/science.1068275

[25] Yu JH, Hamari Z, Han KH, Seo JA, Reyes-Dominguez Y, et al (2002) A draft sequence of the rice genome (*Oryza sativa* L. ssp *indica*). Science 296: 79–92. 11935017 10.1126/science.1068037

[26] D'Souza CA, Heitman J (2001) Conserved cAMP signaling cascades regulate fungal development and virulence. FEMS Microbiol Rev 25: 349–364. 11348689 10.1111/j.1574-6976.2001.tb00582.x

[27] Jeon J, Goh J, Yoo S, Chi MH, Choi J, et al (2008) A putative MAP kinase kinase kinase, *MCK1*, is required for cell wall integrity and pathogenicity of the rice blast fungus, *Magnaporthe oryzae* . Mol Plant Microbe Interact 21: 525–534. 18393612 10.1094/MPMI-21-5-0525

[28] Lee YH, Dean RA (1993) cAMP regulates infection structure formation in the plant-pathogenic fungus *Magnaporthe grisea* . Plant Cell 5: 693–700. 12271080 10.1105/tpc.5.6.693 PMC160306

[29] Rho HS, Jeon J, Lee YH (2009) Phospholipase C-mediated calcium signalling is required for fungal development and pathogenicity in *Magnaporthe oryzae* . Mol Plant Pathol 10: 337–346. 19400837 10.1111/j.1364-3703.2009.00536.x PMC6640429

[30] Xu JR, Hamer JE (1996) MAP kinase and cAMP signaling regulate infection structure formation and pathogenic growth in the rice blast fungus *Magnaporthe grisea* . Genes Dev 21: 2696–2706. 10.1101/gad.10.21.2696 8946911

[31] Park G, Xue GY, Zheng L, Lam S, Xu JR (2002) *MST12* regulates infectious growth but not appressorium formation in the rice blast fungus *Magnaporthe grisea* . Mol Plant Microbe Interact 15: 183–192. 11952120 10.1094/MPMI.2002.15.3.183

[32] DeZwaan TM, Carroll AM, Valent B, Sweigard JA (1999) *Magnaporthe grisea* Pth11p is a novel plasma membrane protein that mediates appressorium differentiation in response to inductive substrate cues. Plant Cell 11: 2013–2030. 10521529 10.1105/tpc.11.10.2013 PMC144101

[33] Thines E, Eilbert F, Sterner O, Anke H (1997) Signal transduction leading to appressorium formation in germinating conidia of *Magnaporthe grisea*: effects of second messengers diacylglycerols, ceramides and sphingomyelin. FEMS Microbiol Lett 156: 91–94.

[34] Jenkins GM, Richards A, Wahl T, Mao CG, Obeid L, et al (1997) Involvement of yeast sphingolipids in the heat stress response of *Saccharomyces cerevisiae* . J Biol Chem 272: 32566–32572. 9405471 10.1074/jbc.272.51.32566

[35] Klose J, de Sa MM, Kronstad JW (1992) Lipid-induced filamentous growth in *Ustilago maydis* . Mol Microbio 52: 823–835. 10.1111/j.1365-2958.2004.04019.x 15101987

[36] Patton JL, Srinivasan B, Dickson RC, Lester RL (1992) Phenotypes of Sphingolipid-dependent strains of *Saccharomyce scerevisiae* . J Bacteriol 174: 7180–7184. 1429441 10.1128/jb.174.22.7180-7184.1992 PMC207409

[37] Shea JM, Del Poeta M (2006) Lipid signaling in pathogenic fungi. Curr Opin Microbiol 9: 352–358. 16798065 10.1016/j.mib.2006.06.003

[38] Spiegel S, Foster D, Kolesnick R (1996) Signal transduction through lipid second messengers. Curr Opin Cell Biol 8: 159–167. 8791422 10.1016/s0955-0674(96)80061-5

[39] Wang ZY, Soanes DM, Kershaw MJ, Talbo NJ (2007) Functional analysis of lipid metabolism in *Magnaporthe grisea* reveals a requirement for peroxisomal fatty acid β-oxidation during appressorium-mediated plant infection. Mol Plant Microbe Interact 20: 475–491. 17506326 10.1094/MPMI-20-5-0475

[40] Wang ZY, Thornton CR, Kershaw MJ, Li DB, Talbot NJ (2003) The glyoxylate cycle is required for temporal regulation of virulence by the plant pathogenic fungus *Magnaporthe grisea* . Mol Microbiol 47: 1601–1612. 12622815 10.1046/j.1365-2958.2003.03412.x

[41] Choi J, Cheong K, Jung K, Jeon J, Lee GW, et al (2013) CFGP 2.0: a versatile web-based platform for supporting comparative and evolutionary genomics of fungi and Oomycetes. Nucleic Acids Res 41: D714–D719. 23193288 10.1093/nar/gks1163 PMC3531191

[42] Stukey J, Carman GM (1997) Identification of a novel phosphatase sequence motif. Protein Sci 6: 469–472. 9041652 10.1002/pro.5560060226 PMC2143653

[43] Jia YJ, Kai M, Wada I, Sakane F, Kanoh H (2003) Differential localization of lipid phosphate phosphatases 1 and 3 to cell surface subdomains in polarized MDCK cells. FEBS Lett 552: 240–246. 14527693 10.1016/s0014-5793(03)00931-1

[44] Horton P, Park KJ, Obayashi T, Fujita N, Harada H, et al (2007) WoLF PSORT: protein localization predictor. Nucleic Acids Res 35: W585–587. 17517783 10.1093/nar/gkm259 PMC1933216

[45] Kim S, Park J, Park SY, Mitchell TK, Lee YH (2010) Identification and analysis of *in planta* expressed genes of *Magnaporthe oryzae* . BMC Genomics 11: 104. 20146797 10.1186/1471-2164-11-104 PMC2832786

[46] Yu JH, Hamari Z, Han KH, Seo JA, Reyes-Dominguez Y, et al (2004) Double-joint PCR: a PCR-based molecular tool for gene manipulations in filamentous fungi. Fungal Genet Biol 41: 973–981. 15465386 10.1016/j.fgb.2004.08.001

[47] Koga H, Dohi K, Nakayachi O, Mori M (2004) A novel inoculation method of *Magnaporthe grisea* for cytological observation of the infection process using intact leaf sheaths of rice plants. Physiol Mol Plant Pathol 64: 67–72.

[48] Torres MA, Jones JDG, Dangl JL (2006) Reactive oxygen species signaling in response to pathogens. Plant Physiol 141: 373–378. 16760490 10.1104/pp.106.079467 PMC1475467

[49] Wojtaszek P (1997) Oxidative burst: An early plant response to pathogen infection. Biochem J 322: 681–692. 9148737 10.1042/bj3220681 PMC1218243

[50] Choi J, Kim KS, Rho HS, Lee YH (2011) Differential roles of the phospholipase C genes in fungal development and pathogenicity of *Magnaporthe oryzae* . Fungal Genet Biol 48: 445–455. 21237279 10.1016/j.fgb.2011.01.001

[51] Ghannoum MA (2000) Potential role of phospholipases in virulence and fungal pathogenesis. Clin Microbiol Rev 13: 122–143. 10627494 10.1128/cmr.13.1.122-143.2000 PMC88936

[52] Rhome R, Del Poeta M (2009) Lipid signaling in pathogenic fungi. Annu Rev Microbiol 63: 119–31. 19450140 10.1146/annurev.micro.091208.073431 PMC5125068

[53] Carrasco S, Merida I (2007) Diacylglycerol, when simplicity becomes complex. Trends Biochem Sci 32: 27–36. 17157506 10.1016/j.tibs.2006.11.004

[54] Talbot NJ, Foster AJ (2001) Genetics and genomics of the rice blast fungus *Magnaporthe grisea*: Developing an experimental model for understanding fungal diseases of cereals. Adv Bot Res 34: 263–287.

[55] Villalba F, Collemare J, Landraud P, Lambou K, Brozek V, et al (2008) Improved gene targeting in *Magnaporthe grisea* by inactivation of *MgKU80* required for non-homologous end joining. Fungal Genet Biol 45: 68–75. 17716934 10.1016/j.fgb.2007.06.006

[56] Sciorra VA, Morris AJ (1999) Roles for lipid phosphate phosphatases in regulation of cellular signaling. Biochim Biophys Acta 1582: 45–51. 10.1016/s1388-1981(02)00136-1 12069809

[57] Goni FM, Alonso A (1999) Structure and functional properties of diacylglycerols in membranes. Prog Lipid Res 38: 1–48. 10396601 10.1016/s0163-7827(98)00021-6

[58] Gomez-Fernandez JC, Corbalan-Garcia S (2007) Diacylglycerols, multivalent membrane modulators. Chem Phys Lipids 148: 1–25. 17560968 10.1016/j.chemphyslip.2007.04.003

[59] Goldston AM, Powell RR, Temesvari LA (2012) Sink or swim: lipid rafts in parasite pathogenesis. Trends Parasitol 28: 417–26. 22906512 10.1016/j.pt.2012.07.002 PMC9721446

[60] Alvarez FJ, Douglas LM, Konopka JB (2007) Sterol-rich plasma membrane domains in fungi. Eukaryot Cell 6: 755–63. 17369440 10.1128/EC.00008-07 PMC1899238

[61] Siafakas AR, Wright LC, Sorrell TC, Djordjevic JT (2006) Lipid rafts in *Cryptococcus neoformans* concentrate the virulence determinants phospholipase B1 and Cu/Zn superoxide dismutase. Eukaryot Cell 5: 488–98. 16524904 10.1128/EC.5.3.488-498.2006 PMC1398056

[62] Park SY, Chi MH, Milgroom MG, Kim H, Han SS, et al (2010) Genetic Stability of *Magnaporthe oryzae* during successive passages through rice plants and on artificial medium. Plant Pathol J 26: 313–320.

[63] Choi J, Park J, Jeon J, Chi MH, Goh J, et al (2007) Genome-wide analysis of T-DNA integration into the chromosomes of *Magnaporthe oryzae* . Mol Microbiol 66: 826–826. 10.1111/j.1365-2958.2007.05918.x PMC2169514 17850257

[64] Chi MH, Park SY, Lee YH (2009) A quick and safe method for fungal DNA extraction. Plant Pathol J 25: 108–111.

[65] Sambrook J, Russell DW (2001) Molecular cloning: a laboratory manual. Cold Spring Harbor: Cold Spring Harbor Laboratory Press.

[66] Valent B, Chumley FG (1991) Molecular genetic analysis of the rice blast fungus, *Magnaporthe grisea* . Annu Rev Phytopathol 29: 443–467. 18479196 10.1146/annurev.py.29.090191.002303

[67] McGinnis S, Madden TL (2004) BLAST: at the core of a powerful and diverse set of sequence analysis tools. Nucleic Acids Res 32: W20–W25. 15215342 10.1093/nar/gkh435 PMC441573

[68] Thompson JD, Higgins DG, Gibson TJ (1994) CLUSTAL W: improving the sensitivity of progressive multiple sequence alignment through sequence weighting, position-specific gap penalties and weight matrix choice. Nucleic Acids Res 22: 4673–4680. 7984417 10.1093/nar/22.22.4673 PMC308517

[69] von Heijne G (1992) Membrane protein structure prediction. Hydrophobicity analysis and the positive-inside rule. J Mol Biol 225: 487–94. 1593632 10.1016/0022-2836(92)90934-c

