# Supplementary figures and images for: Regulation of Cellular Diacylglycerol through Lipid Phosphate Phosphatases Is Required for Pathogenesis of the Rice Blast Fungus, Magnaporthe oryzae
Source: PLoS One. 2014 Jun 24;9(6):e100726. doi: 10.1371/journal.pone.0100726 (PMC4069076; doi:10.1371/journal.pone.0100726)

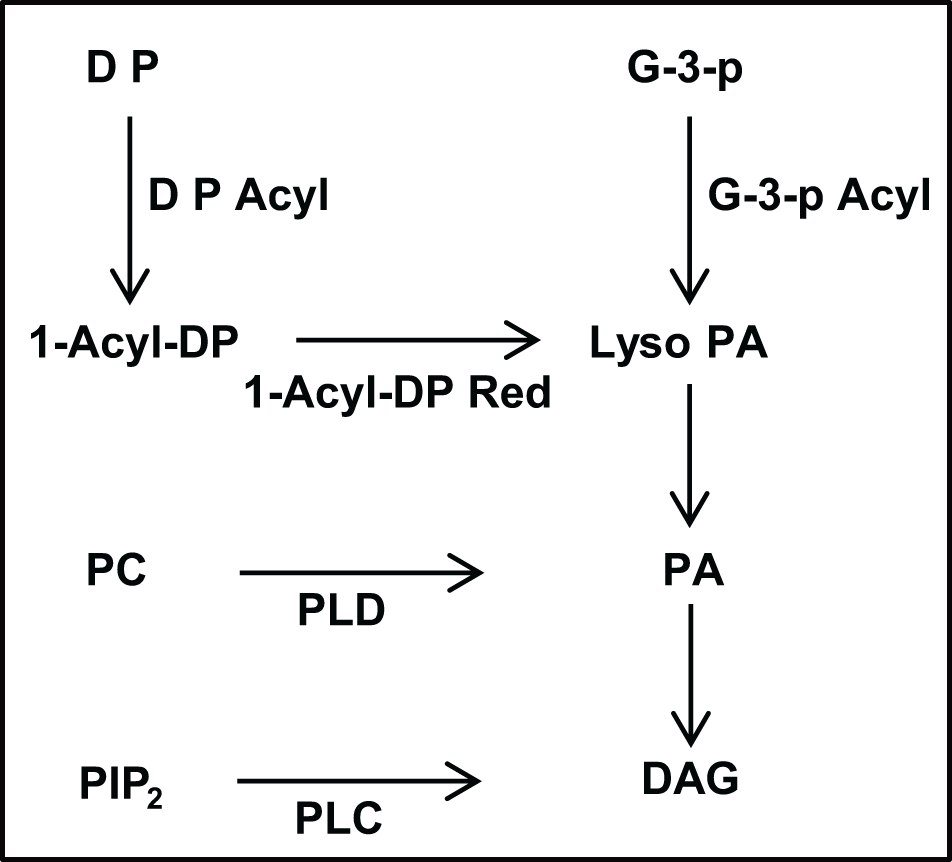

Supplement: Figure S1 — The enzymatic reactions leading to DAG generation through different pathway. D P, dihydroxyaceton phosphate; D P Acyl, dihydroxyaceton phosphate acyltransferase; 1-Acyl-D P, 1-Acyl-dihydroxyaceton phosphate; 1-Acyl-D P Red, 1-Acyl-dihydroxyaceton phosphate reductase; G-3-p, glycerol-3-phosphate; G-3-p Acyl, glycerol-3-phosphate acyltransferase; Lyso PA; lysophosphatidic acid; PC, phosphatidylcholine; PLD, phospholipase D; PA, phosphatidic acid; PIP2, phosphatidyl inositol-4-5-bisphosphate; PLC, phospholipase C; DAG, diacylglycerol. (TIF) [file pone.0100726.s001.tif]

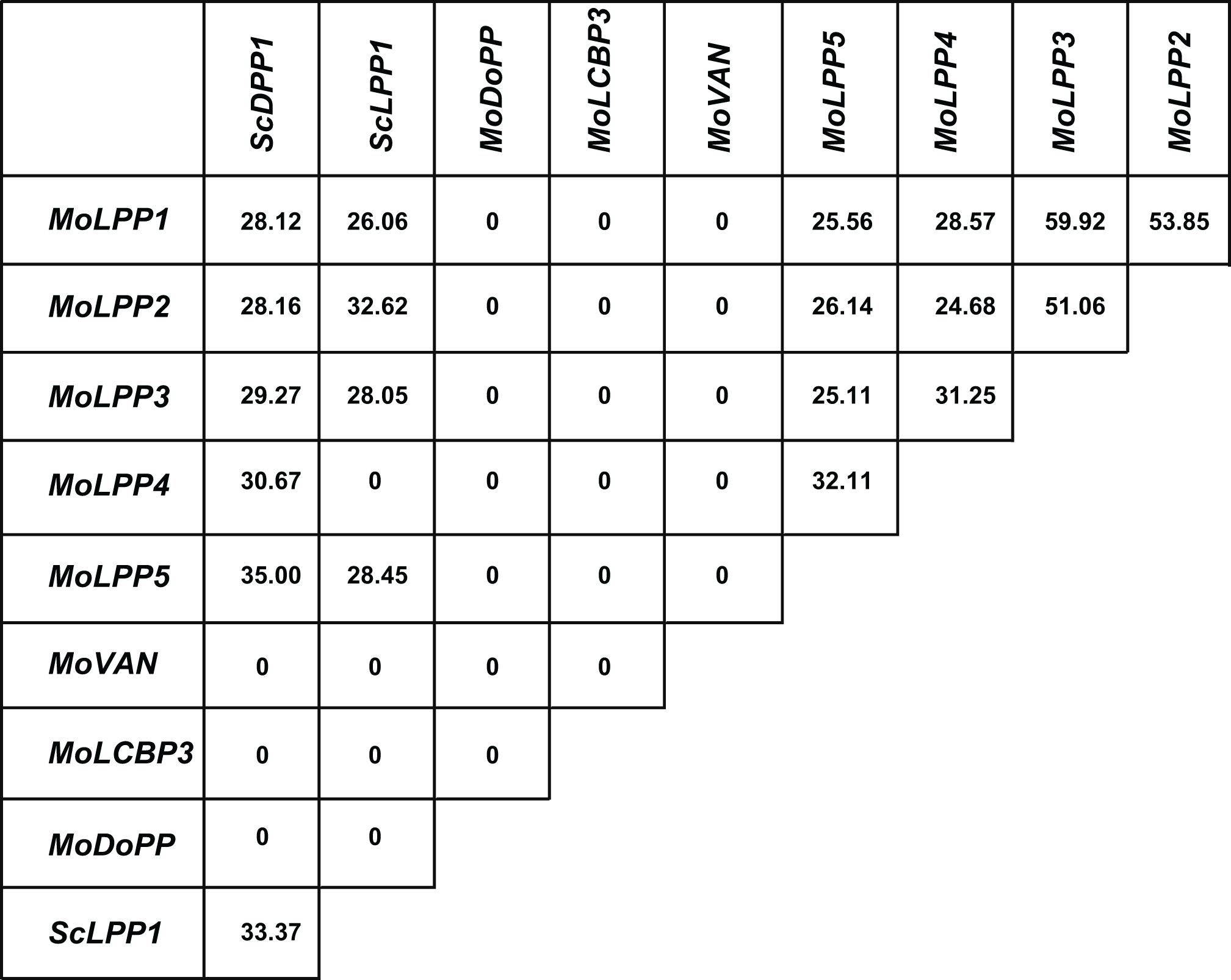

Supplement: Figure S2 — Sequence similarities of PAP2 domain of eight genes with each other and also with the yeast ScLPP1 and ScDPP1 . Sequence similarities were measured by using BLAST2 with the amino acid sequences provided at Comparative Fungal Genomics Platform (CFGP), (http://cfgp.snu.ac.kr/). (TIF) [file pone.0100726.s002.tif]

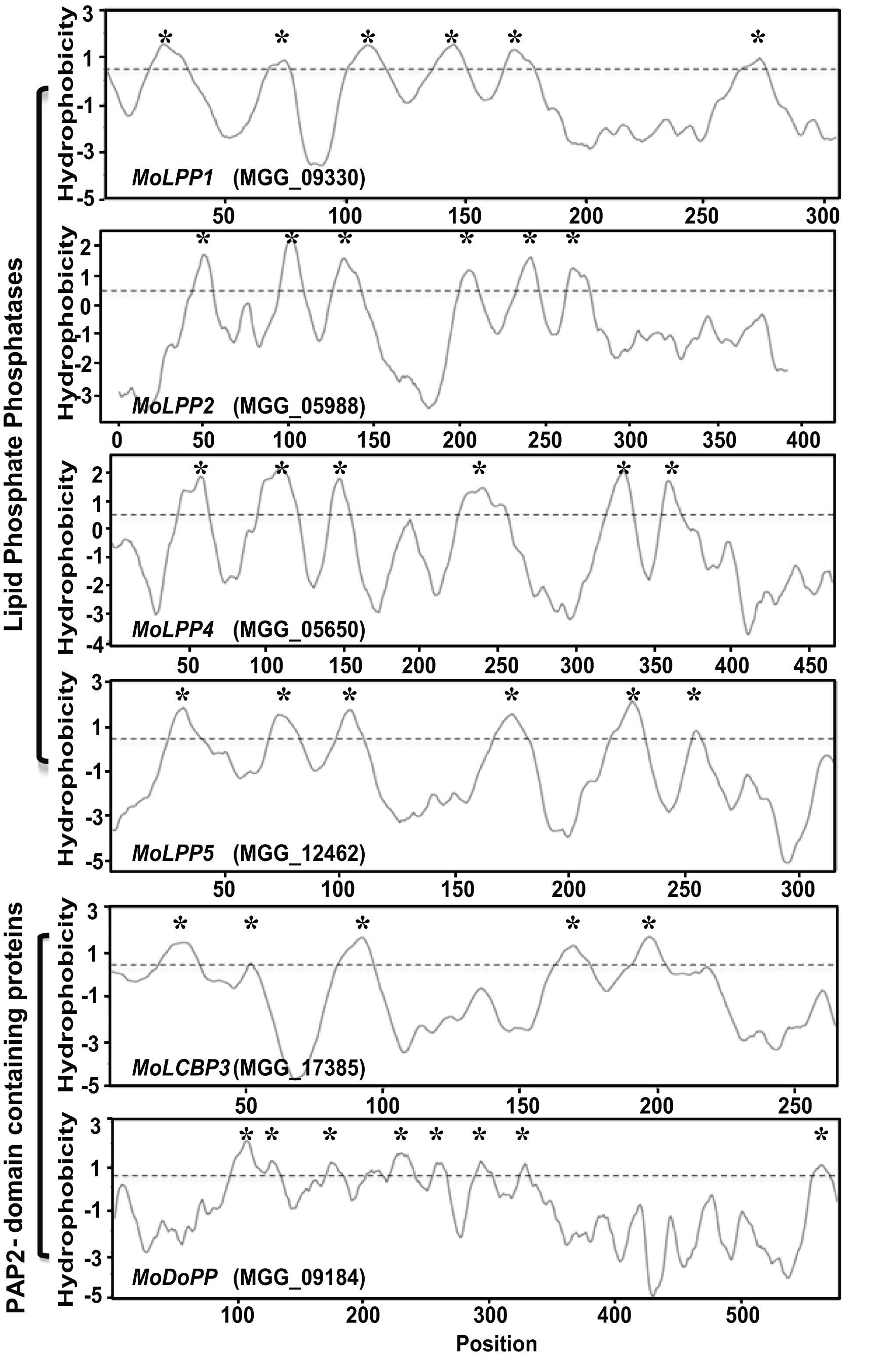

Supplement: Figure S3 — Hydropathy plots of the deduced MoLPP1 , MoLPP2 , MoLPP4 , MoLPP5 , MoLCBP3 and MoDoPP proteins showing potential transmembrane domains ( TM1-6 ). Asterisks are indicating the potential number of membrane-spanning domains. Hydropathy plot was generated using TopPred 2 (http://www.sbc.su.se/~erikw/toppred2/). Cutoff value (0.6) was indicated by dotted horizontal line. (TIF) [file pone.0100726.s003.tif]

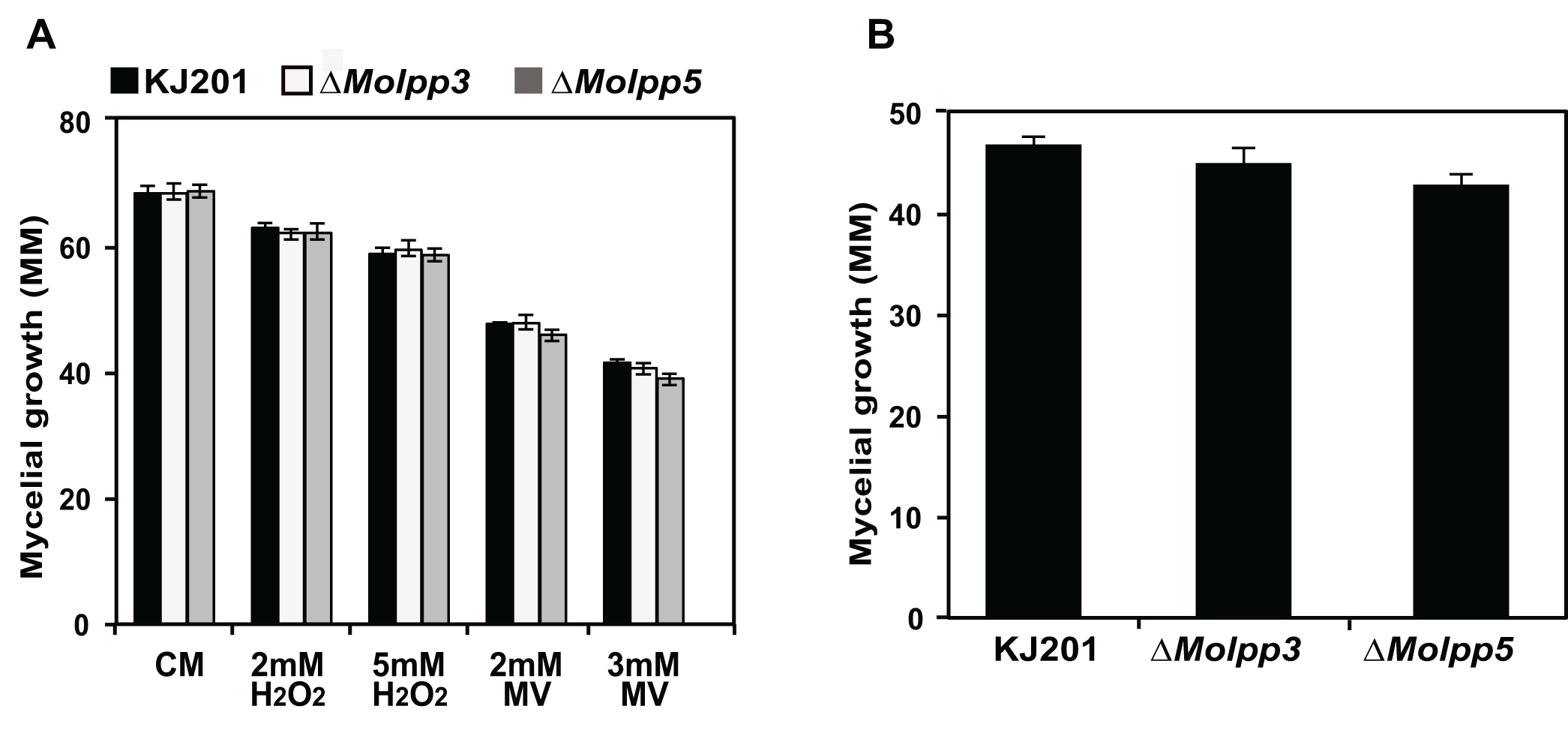

Supplement: Figure S4 — Mycelial growth in different stress conditions. (A) Oxidative stress (B) Congo red. Mycelial block was inoculated in CM agar plate containing different concentration of hydrogen peroxide, methyl viologen and congo red. Data was taken at 10 dpi with three independent experiments with three replications. (TIF) [file pone.0100726.s004.tif]

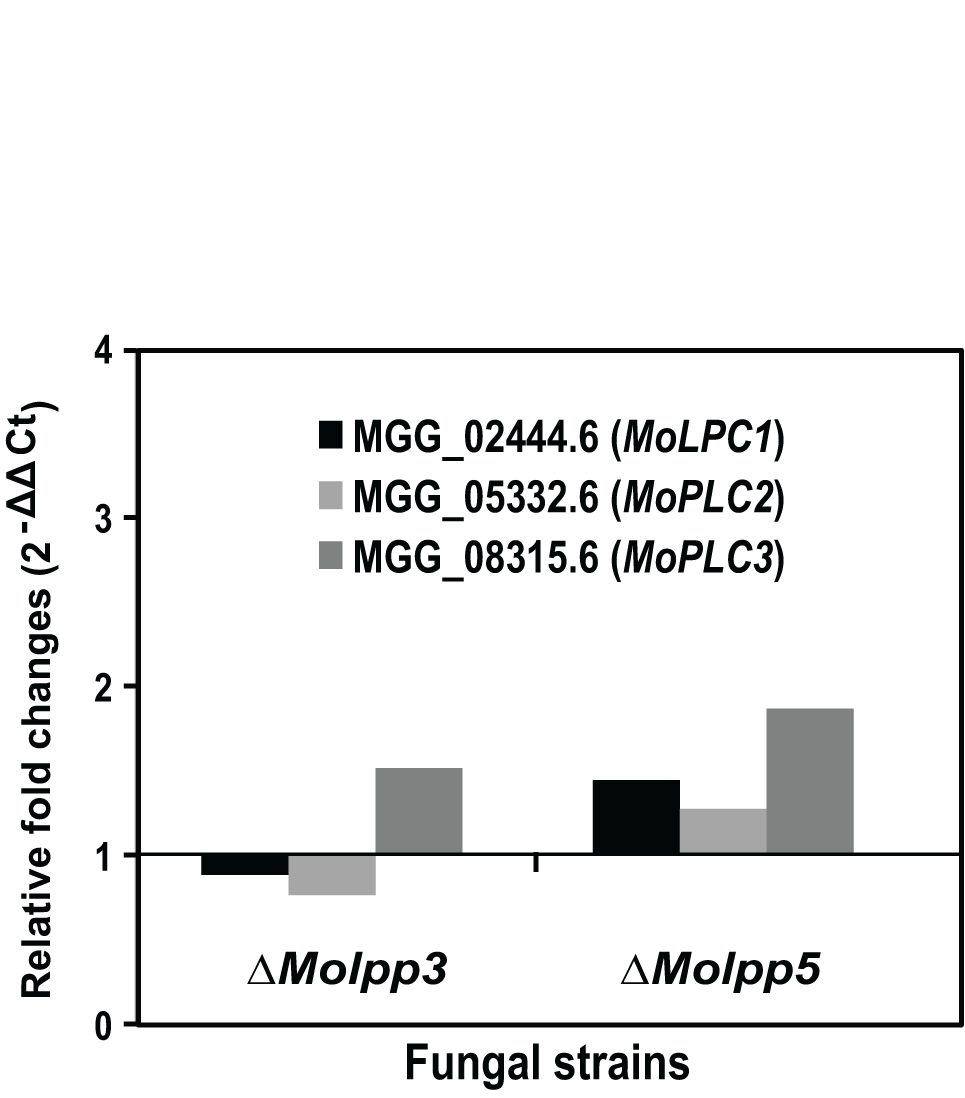

Supplement: Figure S5 — Transcriptional profiling of phospholipase genes of M. oryzae in ΔMolpp3 and ΔMolpp5 knockout mutants. Transcriptional expressions of MoPLC genes in knock-out mutants were compared with the expression of wild type strain following normalization using β-tubulin. (TIF) [file pone.0100726.s005.tif]
