# Supplementary material for: Regulation of Cellular Diacylglycerol through Lipid Phosphate Phosphatases Is Required for Pathogenesis of the Rice Blast Fungus, Magnaporthe oryzae
Source: PLoS One. 2014 Jun 24;9(6):e100726. doi: 10.1371/journal.pone.0100726 (PMC4069076; doi:10.1371/journal.pone.0100726)
Supplement: Table S1 — Oligo sequences used in this study. (DOCX) [file pone.0100726.s006.docx]

Table S1. Oligo sequences used in this study.

| **Primer name** | **Sequence (5’ → 3’)** |
| --- | --- |
| **For generation and confirmation of *MoLPP* (*1-5*) mutants:** | |
| MoLPP1_UF | ACCTTGCTTACGAGTACG |
| MoLPP1_UR | TGTGTGACGCAGCTTCTT |
| MoLPP1_DF | GATAGGTAAAGACAGTGGGG |
| MoLPP1_DR | ATCAGGAGAGATAGCTCG |
| MoLPP1_NF | GACTTGTCAGGACTCTTG |
| MoLPP1_NR | ACTACTTGTAGAACCCCG |
| MoLPP2_UF | CAGCGCCATCACGATTAT |
| MoLPP2_UR | CAGAAGACCAATGTCTGGT |
| MoLPP2_DF | ACAGTCGAGGTCTTGGTA |
| MoLPP2_DR | ACAACCTGCCCAACAT |
| MoLPP2_NF | CCGTCAAAGTGTTTGTCG |
| MoLPP2_NR | ATGCCACCAAGAAGTACC |
| MoLPP3_UF | TTCGAAGGTGCCAAGT |
| MoLPP3_UR | GCAGCTTGATCCACAA |
| MoLPP3_DF | AGCAACGACTGTACGA |
| MoLPP3_DR | TTTGGGCGAATCACAG |
| MoLPP3_NF | CCAACCTGCTTTCCAT |
| MoLPP3_NR | AAGCACGCTTACCCTTA |
| MoLPP4_UF | TTCGTCAGCAACTAGAGG |
| MoLPP4_UR | GTATTACATGTAGGAGCC |
| MoLPP4_DF | TTGCTAGAGCTCTCCTGT |
| MoLPP4_DR | GGATTTCAAAGGGGGGTT |
| MoLPP4_NF | TGATGGGGGAAAATCAGG |
| MoLPP4_NR | TCTAAACGAACGCTCGGA |
| MoLPP5_UF | ATCGAGGATCTCTTGACG |
| MoLPP5_UR | TCCGCGTATTGCTTTC |
| MoLPP5_DF | TTG CGA GAC GTC TTG A |
| MoLPP5_DR | CTCCTTCACTGTAGGCAA |
| MoLPP5_NF | TGCGGATATCCAGTGATG |
| MoLPP5_NR | AATGCCAACCCACCAA |
| HPH_F | GGCTTGGCTGGAGCTAGTGGAGG |
| HPH_R | GTTGGTGTCGATGTCAGCTCCGGAG |
| **For qRT-PCR** | |
| MoLPP1_qRT-F | GGAGAAGCGAAGAGGTGTTT |
| MoLPP1_qRT-R | GGCGTTCAGATACAGATAGAGG |
| MoLPP2_qRT-F | TCATCGGAATTCTGTACTCGC |
| MoLPP2_qRT-R | ACAAACGTCGAGGAAGTGAG |
| MoLPP3_qRT-F | TCCTCAGCACCTTTTGTCAG |
| MoLPP3_qRT-R | GTAGCCAATGCCCTCTGTG |
| MoLPP4_qRT-F | GACATCTCCCGTCCCAATG |
| MoLPP4_qRT-R | AGATGAATATGGCACCGAAGG |
| MoLPP5_qRT-F | CAGCTTAGGATATTCGCCTACG |
| MoLPP5_qRT-R | CACCCTCTCCATCTTTCCAG |
| MoPLC1_qRT-F | GCTCCAACCGACATCTACATC |
| MoPLC1_qRT-R | TTATTGACTACCGCCATCGAC |
| MoPLC2_qRT-F | GATGCGAACTTATCCCTCTGG |
| MoPLC2_qRT-R | CCTGCAAACATCCCTTCATTG |
| MoPLC3_qRT-F | CTCATCCTTCATCCCCTACG |
| MoPLC3_qRT-R | CCCGTGCTCTTTATAGTGGTAC |
| Β-tub_qRT-F | TCGACAGCAATGGAGTTTAC |
| Β-tub_qRT-R | AGCACCAGACTGACCGAAGA |
